# Type 2 diabetes and pre-diabetes are associated with obstructive sleep apnea in extremely obese subjects: A cross-sectional study

**DOI:** 10.1186/1475-2840-10-84

**Published:** 2011-09-25

**Authors:** Jan Magnus Fredheim, Jan Rollheim, Torbjørn Omland, Dag Hofsø, Jo Røislien, Kristian Vegsgaard, Jøran Hjelmesæth

**Affiliations:** 1 Morbid Obesity Centre, Department of Medicine, Vestfold Hospital Trust, 3103 Tønsberg, Norway; 2 Department of Otolaryngology - Head and Neck Surgery, Vestfold Hospital Trust, 3103 Tønsberg, Norway; 3 Department of Medicine, Akershus University Hospital, Lørenskog, Norway; 4 Department of Biostatistics, Institute of Basic Medical Sciences, University of Oslo, 0317 Oslo, Norway

**Keywords:** Obstructive sleep apnea, type 2 diabetes, prediabetes, oral glucose tolerance test, inflammation

## Abstract

**Background:**

Obstructive sleep apnea (OSA) is a common yet underdiagnosed condition. The aim of our study is to test whether prediabetes and type 2 diabetes are associated with obstructive sleep apnea (OSA) in extremely obese (BMI ≥ 40 kg/m^2^) subjects.

**Methods:**

One hundred and thirty seven consecutive extremely obese patients (99 females) from a controlled clinical trial [MOBIL-study (Morbid Obesity treatment, Bariatric surgery versus Intensive Lifestyle intervention Study) (ClinicalTrials.gov number NCT00273104)] underwent somnography with Embletta^® ^and a 2-hour oral glucose tolerance test (OGTT). OSA was defined by an apnea-hypopnea index (AHI) ≥ 5 events/hour. Patients were categorized into three groups according to criteria from the American Diabetes Association: normal glucose tolerance, pre-diabetes and type 2 diabetes. Multiple logistic regression analysis was used to identify possible determinants of OSA.

**Results:**

The patients had a mean (SD) age of 43 (11) years and a body mass index (BMI) of 46.9 (5.7) kg/m^2^. Males had significantly higher AHI than females, 29 (25) vs 12 (17) events/hour, p < 0.001. OSA was observed in 81% of men and in 55% of women, p = 0.008. Twenty-nine percent of subjects had normal glucose tolerance, 42% had pre-diabetes and 29% had type 2 diabetes. Among the patients with normal glucose tolerance 33% had OSA, while 67% of the pre-diabetic patients and 78% of the type 2 diabetic patients had OSA, p < 0.001. After adjusting for age, gender, BMI, high sensitive CRP and HOMA-IR, both pre-diabetes and type 2 diabetes were still associated with OSA, odds ratios 3.18 (95% CI 1.00, 10.07), p = 0.049 and 4.17 (1.09, 15.88), p = 0.036, respectively. Mean serum leptin was significantly lower in the OSA than in the non-OSA group, while other measures of inflammation did not differ significantly between groups.

**Conclusions:**

Type 2 diabetes and pre-diabetes are associated with OSA in extremely obese subjects.

**Trial registration:**

MOBIL-study (Morbid Obesity treatment, Bariatric surgery versus Intensive Lifestyle intervention Study) (ClinicalTrials.gov number NCT00273104)

## Introduction

Obstructive sleep apnea (OSA) is an under-diagnosed yet common disease [1] which is associated with increased incidence of cardiovascular disease and substantially increased risk of death [2,3]. There is a strong relationship between OSA and obesity [4,5], indeed, approximately 70% of patients with OSA are obese [6]. Obesity related subcutaneous and periluminal fat deposits may alter compliance of upper airway walls and narrow the luminal area, thus increasing the likelihood of airway collapse when exposed to the intraluminal negative pressure caused by inspiration [5].

OSA is also associated with increased risk of type 2 diabetes (T2DM) [7,8]. Several mechanisms, including intermittent hypoxia, sleep fragmentation and immune activation may contribute to this association [8-11]. Both adipose tissue and diabetes are associated with immune activation and subsequent increase in the circulating concentration of pro-inflammatory cytokines [12,13], which in turn may play a role in the pathogenesis of OSA.

All dilator muscles of the upper airways are innervated by the vagal nerve, with patency of the upper airway during sleep depending on well functioning nerve, neuromuscular junction and muscle. Diabetes increases the risk of neuropathy in the autonomic nervous system as well as in the extremities [14]. Impaired vagal activity may lead to dysfunction of upper airway muscles and increased risk of OSA [15].

Visceral obesity, high insulin levels and insulin resistance have been associated with increased risk of OSA [16,17]. Some studies have shown a high prevalence of T2DM and pre-diabetes (preDM) in obese subjects with OSA [17-20], indicating that OSA may cause glucose intolerance [17].

We aimed to investigate whether extremely obese subjects with T2DM and preDM have higher odds of OSA than their counterparts with normal glucose tolerance.

## Methods

### Study design

This is a pre-defined, cross-sectional analysis of baseline data from a controlled clinical trial [MOBIL-study (Morbid Obesity treatment, Bariatric surgery versus Intensive Lifestyle intervention Study) (ClinicalTrials.gov number NCT00273104)] [21]. The study design and setting have previously been described in detail [22]. A brief summary of materials and methods is given below.

### Setting/Participants

**Figure 1:**
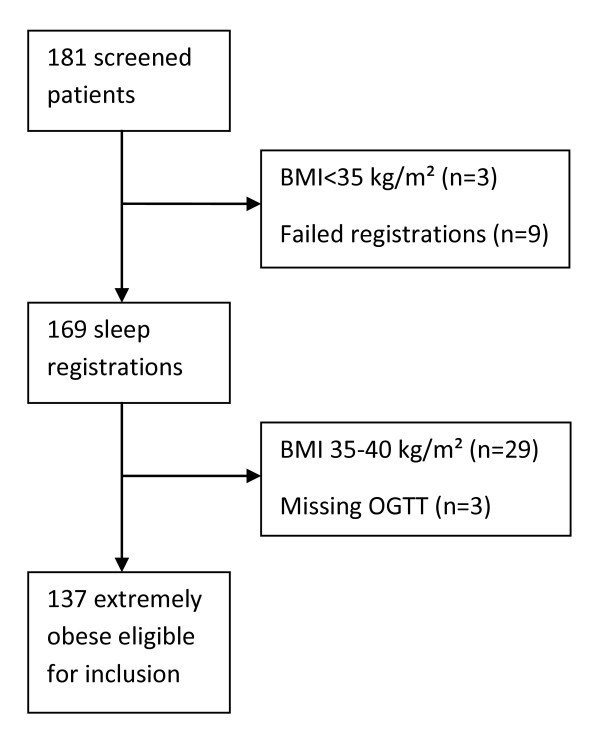
**Flow chart of 181 patients screened for participation in the MOBIL-study**. Initially three patients were excluded due to a BMI < 35 kg/m² and nine due to failed sleep registrations. Twenty nine of these patients had a BMI < 40 kg/m² and three had a missing OGTT, thereby leaving 137 extremely obese patients for inclusion in the present analysis.

All participants were recruited from the Morbid Obesity Centre, Vestfold Hospital Trust, Tønsberg, Norway. The study protocol had the approval of the regional ethics committee and all patients provided written informed consent. Only patients with extreme obesity (obesity grade III; BMI ≥ 40 kg/m²) were included in the present study. Of the 181 patients screened for participation in the MOBIL-study [21], 35 patients were excluded due to a BMI < 40 kg/m² (n = 32) or a missing oral glucose tolerance test (OGTT; n = 3). After the exclusion of an additional nine patients who failed to comply with sleep registrations, 137 extremely obese patients (101 females) were included in the present analysis (figure 1).

### Variables and definitions

The primary outcome variable was OSA, which was defined as at least five apneas or hypopneas, lasting more than ten seconds, per hour. The main explanatory variable was glucose tolerance (categorised as normal glucose tolerance (NGT), preDM and T2DM) according to the American Diabetes Association classificiation 2010 [23]). Other explanatory variables and possible confounders were age, gender, anthropometric measures, smoking, alcohol consumption, hypertension, relevant medication, insulin resistance (as measured by HOMA-IR) and high sensitivity CRP (hsCRP). Arterial hypertension was defined by systolic blood pressure ≥ 140 mmHg, diastolic blood pressure ≥ 90 or the use of antihypertensive medication. Microalbuminuria was defined as albumin-creatinine ratio ≥ 3 mg/mmol and < 30 mg/mmol, and manifest proteinurea as albumin-creatinine ratio ≥30 mg/mmol.

A computer based homeostasis model assessment of insulin resistance (HOMA-IR) was used, HOMA Calculator v2.2.2 [24].

An apnea was defined as a 90% or more reduction of baseline nasal air flow lasting at least ten seconds. Hypopneas were defined as a 50% - 90% decrease in pre-event baseline of nasal air flow lasting ≥ 10 seconds accompanied by at least a 3% drop in oxygen saturation and/or signs of awakening or increased stress. OSA was defined as having an apnea-hypopnea index (AHI) ≥ 5 events/hour. Mild OSA was defined as AHI 5-15, moderate 15-30 and severe 30 or more events/hour. Scoring rules were in accordance with the American Academy of Sleep Medicine manual for scoring of sleep from 2007 [25].

### Data sources/measurement

The reference standard method of sleep registration is polysomnography, whereby patients are admitted and monitored during an entire night's sleep. This method is, however, both time consuming and expensive, and removes the patient from his/her natural sleep environment. Portable monitors, on the other hand, can be used at home and are simple to function. This enables the patient to maintain normal sleep environment and pre-bed rituals [26]. The accuracy of home sleep diagnostic systems like the Embletta™ is considered to be sufficient for most patients in the diagnosis of OSA [27,28]. The night to night variability of sleep-disordered breathing is low, and a retrospective study performed by Stepnowsky et al suggests that one nights recording is sufficient to diagnose OSA in nine out of ten cases [29].

The sleep registrations were performed using Embletta™, a portable multi-channel recorder consisting of a nasal cannula, two piezoelectric belts, a finger pulse oximeter and a body position detector [28]. The two piezoelectric belts were placed around the thorax and abdomen to monitor respiratory movements. To avoid inter-rater variation, Embletta™ recordings were manually scored by the same person [30]. In a study of a large dataset the mean epoch by epoch agreement between scorers for all records was 73% (range 67-82%) [31].

The equipment assembly included both written and oral instructions. The patients equipped themselves prior to going to bed and the registrations were scored the next day. Treatment was provided according to current guidelines [32]. Patients using continuous positive airway pressure prior to the study had a one week wash-out period during which they did not use the machine. All patient data was registered in a Case Report Form (CRF).

All patients, except those with drug-treated T2DM, underwent a standardised OGTT [33]. The patients were categorised into three groups: NGT; fasting plasma glucose <5.6 mmol/L and/or 2-hour plasma glucose <7.8 mmol/L, preDM; fasting plasma glucose 5.6 - 6.9 mmol/L and/or 2-hour plasma glucose 7.8 - 11.0 mmol/l or T2DM; fasting plasma glucose ≥ 7.0 mmol/L or 2-hour plasma glucose ≥ 11.1 mmol/L. In addition, patients with either preDM or T2DM were categorised as to their having abnormal glucose tolerance in supplementary analyses.

### Statistical methods

Data are given as either mean (SD) or proportions (%) unless stated otherwise. Between group differences were assessed using independent samples t-test or analysis of variance (ANOVA) (continuous data) and Fisher's exact test (categorical data). Skewed variables were transformed using natural logarithms before statistical analysis. Multiple logistic regression analyses with pre-defined explanatory variables and OSA (yes/no, cut off AHI = 5) as the dependent variable were also performed [25]. We fitted one crude (unadjusted) logistic regression model (model 1) and three separate multiple logistic regression models (models 2-4). In model 1 glucose tolerance status as a categorical variable was entered as the sole explanatory variable. In model 2 we adjusted for established confounding factors; gender, age and BMI (waist circumference, neck circumference and waist-to-hip ratio were substituted for BMI in supplementary analyses). In model 3 HOMA-IR was added to model 2 to address the possible modifying effect of insulin resistance. In model 4, in order to assess the possible impact of inflammation, hsCRP was added to model 3. In supplementary analyses glucose tolerance as a categorical variable was replaced with HbA1c. The multiple logistic regression analysis was repeated in all models using AHI cut off 15: the recommended clinical cut off for initializing CPAP treatment [32].

To address the issue of multicollinearity we performed calculations of Spearman correlation between categories of glucose tolerance and both HOMA-IR and hsCRP. In addition we assessed the variance inflation factor (VIF) in the logistic regression models. The analyses were performed using SPSS 16.0 (SPSS, Chicago, IL).

## Results

**Figure 2:**
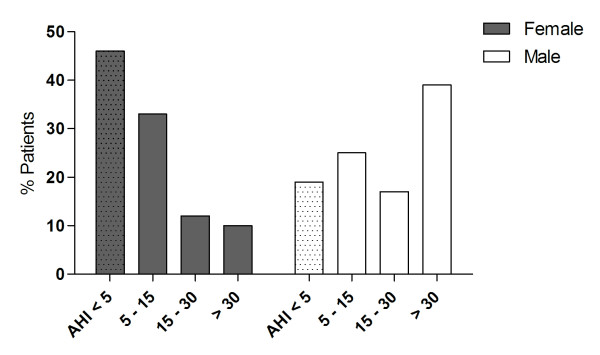
**Categories of morbidly obese subjects according to various levels of the apnea-hypopnea index (AHI)**. Dotted bars represent patients without OSA (AHI < 5 events/hour). Mild OSA is defined as AHI 5 - 15 events/hour, moderate OSA as 15 - 30 events/hour and severe OSA as > 30 events/hour.

A total of 42 (31%) patients had mild OSA, 18 (13%) moderate OSA, and 24 (18%) severe OSA. The prevalence of OSA was higher in men (81%) than in women (55%), p = 0.006, with the severity of OSA more pronounced in males than in females, chi square test p < 0.001 (figure 2). In addition, the prevalence of OSA was significantly higher among postmenopausal (26 out of 29, 90%) than premenopausal women (29 out of 72, 40%), p < 0.001.

Demographic, clinical and biochemical characteristics of the 137 extremely obese subjects, according to the presence or absence of OSA, are shown in table 1. Patients with OSA were significantly older, and they had longer neck circumference, higher waist-to-hip ratios and a higher prevalence of comorbid conditions than those without OSA. There were no significant differences between groups regarding smoking and alcohol consumption (table 1).

**Table 1:** Anthropometric data and comorbidities in 137 extremely obese subjects according to presence or absence of obstructive sleep apnea

Variables	All participants	OSA no	OSA yes	p-value
N	137	53 (39%)	84 (61%)	
Gender (male/female)	36/101 (26/74%)	7/46 (19/45%)	29/55 (81/55%)	0.006
Age (years)	43 (11)	36 (8.8)	48 (9.8)	<0.001
Smokers	36 (26%)	15 (28%)	21 (25%)	0.830
Alcohol consumption (units/week)	1.1 (1.8)	0.9 (1.5)	1.2 (2.1)	0.350
**Anthropometric measures**				
BMI (kg/m²)	46.9 (5.7)	46.3 (5.2)	47.2 (6.1)	0.377
Weight (kg)	136 (22.2)	134 (22.3)	137 (22.1)	0.324
Neck (cm)	42 (4.2)	40.5 (3.8)	43.1 (4.1)	<0.001
Waist (cm)	135 (14)	132 (13.6)	136 (13.5)	0.097
Hip (cm)	139 (12)	140 (10.0)	138 (13.2)	0.389
Waist-to-hip ratio	0.98 (0.09)	0.9 (0.1)	1.0 (0.1)	0.005
**Blood pressure (mean value, 24-hour ambulatory pressure)**				
Systolic (mmHg)	135 (17)	128 (14)	139 (18)	<0.001
Diastolic(mmHg)	84 (10)	81 (10)	86 (10)	0.016
**Comorbidities**				
Coronary heart disease	5 (4%)	0 (0%)	5 (6%)	0.156
Hypertension	48 (35%)	10 (19%)	38 (45%)	0.002
Albuminuria				
Microalbuminuria	19 (14%)	4 (8%)	15 (19%)	0.126
Macroalbuminuria	4 (3%)	0 (0%)	4 (5%)	0.153
Hypothyreosis	18 (13%)	6 (11%)	12 (14%)	0.796
Anxiety and/or depression	56 (41%)	28 (53%)	28 (33%)	0.032
Asthma	35 (26%)	16 (30%)	19 (23%)	0.421
Chronic obstructive pulmonary disease	5 (4%)	2 (4%)	3 (4%)	1.000
**Inflammation**				
Leptin (microg/l)	60.9 (19.3)	66.6 (16.4)	57.4 (20.3)	0.001
Visfatin (ng/ml)	26.0 (63.2)	33.2 (97.6)	21.5 (23)	0.692
High sensitive CRP (mg/l)	3.0 (2.6)	3.5 (3.2)	2.6 (2.1)	0.231
Osteoprotegerin (microg/ml)(	2644 (1640)	2381 (1413)	2809 (1757)	0.099
Adiponectin (pg/ml)	5510 (3368)	5278 (2633)	5656 (3767)	0.954
IL1Ra (pg/ml)	964 (1964)	877 (1913)	1020 (2004)	0.396
Leptin:adiponectin ratio (ng/ml:pg/ml)	0.016 (0.012)	0.017 (0.012)	0.015 (0.012)	0.096

Variables are given as either mean (SD) or proportions (%). Statistical analysis: Fisher's exact test (categorical data), independent samples t-test (continuous data) and Mann-Whitney U test (non-parametric, continuous data).

The prevalence of albuminuria was higher in the OSA group than in the non-OSA group. Mean serum leptin was significantly lower in the OSA than in the non-OSA group, while other measures of inflammation did not differ significantly between groups.

The prescription of analgesics, psychofarmacological drugs, antidepressants, sleep medications and medication for asthma or chronic pulmonary disease did not differ between groups (data not shown). In contrast, the proportion of patients using antihypertensive drugs was higher in the OSA-group (34%) than in the non-OSA group (13%), p = 0.009.

Sleep registration data and measures of glucose metabolism of the 137 extremely obese are presented in table 2. Fasting serum glucose, post-challenge serum glucose and HbA1c were significantly higher in the OSA group than in the non-OSA group. There was no significant difference between the two groups regarding s-insulin and HOMA-IR.

**Table 2:** Sleep registration data and glucose metabolism characteristics in 137 extremely obese subjects according to the presence or absence of obstructive sleep apnea (OSA)

Variables	All participants	OSA no	OSA yes	p-value
N	137	53 (39%)	84 (61%)	
**Sleep registration**				
Apnea-Hypopnea index	16 (20)	2 (2)	25 (22)	<0.001
Oxygen desaturation index	17 (18)	3 (3)	26 (21)	<0.001
Snoring (5 of sleep time)	19 (21)	11 (14)	23 (23)	<0.001
SpO2 (%)	93 (3)	95 (2)	93 (3)	<0.001
**Glucose metabolism**				
Glucose, fasting (mmol/l)	6.6 (2.0)	5.8 (1.1)	7.1 (2.3)	<0.001
Glucose, 2 hour (mmol/l)	7.6 (3.2)	6.5 (2.5)	8.3 (3.4)	0.001
HbA1 (%)	5.9 (1.1)	5.5 (0.8)	6.1 (1.2)	0.001
Insulin (pmol/l)	201 (89)	193 (78)	207 (96)	0.468
HOMA Insulin Resistance	3.8 (1.7)	3.6 (1.4)	4.0 (1.8)	0.178

Variables are given as either mean (SD) or proportions (%). Statistical analysis: Fisher's exact test (categorical data) and independent samples t-test (continuous data).

### OSA according to category of glucose tolerance

A total of 39 (29%) subjects had NGT, 58 (42%) preDM and 40 (29%) T2DM. Among the patients with NGT 33% had OSA, while 67% of the preDM patients and 78% of the T2DM patients had OSA, p = 0.001 and p < 0.001, respectively. The distribution of glucose tolerance categories among different OSA severity categories is shown in table 3.

**Table 3:** Prevalence of various categories of glucose tolerance according to the presence and severity of obstructive sleep apnea in 137 extremely obese subjects

Glucose tolerance status	Non OSA (AHI < 5)	Mild OSA (AHI 5-15)	Moderate OSA (AHI 15-30)	Severe OSA (AHI > 30)
Normal glucose tolerance	49% (26)	10% (4)	17% (3)	25% (6)
Prediabetes	34% (18)	52% (22)	50% (9)	38% (9)
Type 2-diabetes	17% (9)	38% (16)	33% (6)	38% (9)

**Figure 3:**
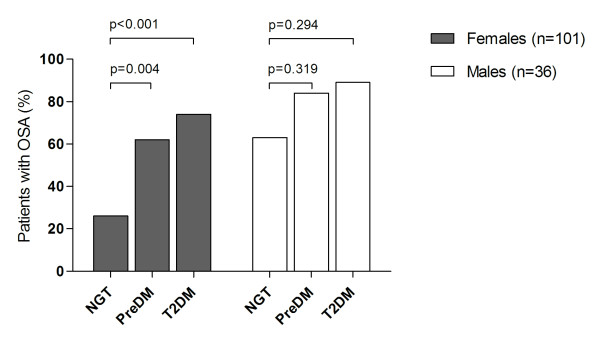
**Prevalence of OSA in 137 extremely obese subjects (101 females) according to various categories of glucose tolerance**.

**Figure 4:**
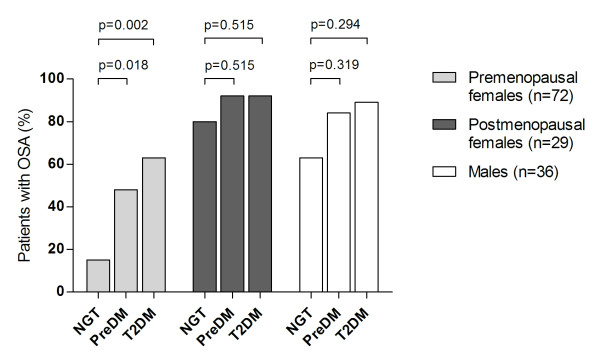
**Prevalence of OSA in 137 extremely obese subjects (101 females) according to various categories of glucose tolerance**. Females are subgrouped according to menopausal status. The mean (SD) ages of men, premenopausal- and postmenopausal women were 44 (11), 38 (8) and 57 (7) years, respectively.

The proportion of patients with OSA was significantly higher in female patients with preDM or T2DM than in those with NGT, p = 0.004 and p < 0.001, respectively (figure 3). These differences were particularly pronounced in premenopausal women (figure 4).

BMI did not differ significantly between the various glucose tolerance groups: NGT-group mean BMI (SD) 46.5 (4.7) kg/m², preDM-group 47.9 (6.8) kg/m² and the T2DM-group 45.9 (4.8) kg/m², p = 0.220. In contrast, mean age increased with worsening glucose tolerance: NGT-group 39 (11) years, preDM-group 43 (10) years and T2DM-group 46 (5) years, respectively, p < 0.001.

### Association between OSA and various measures of glucose tolerance

In a crude, unadjusted logistic regression model (model 1) subjects with preDM and T2DM had approximately 4- and 6-fold increased odds of OSA compared with the normoglycemic group (Table 4). The odds of OSA in preDM and T2DM were attenuated after adjustment for gender, age and BMI (model 2), but remained statistically significant. The substitution of BMI with other anthropometric measures in model 2 did not significantly change the association between abnormal glucose tolerance and OSA (data not shown). Finally, both preDM and T2DM remained associated with significantly higher odds of OSA after further adjustments for HOMA-IR and high sensitive CRP (model 4). Gender and age were both strong predictors of having OSA with ORs (95% CI) of 4.2 (1.2, 14.4) and 1.15 (1.08, 1.21), respectively.

**Table 4:** Odds of obstructive sleep apnea (AHI cut off 5) in extremely obese subjects with type 2-diabetes or prediabetes

	Model 1 OR (95% CI)	Model 2 OR (95% CI)	Model 3 OR (95% CI)	Model 4 OR (95% CI)
Prediabetes	4.4 (1.9-10.6) ^a^	3.3 (1.1-9.4) ^c^	3.2 (1.0-10.1) ^c^	4.0 (1.2-13.2) ^c^
Type 2-diabetes	6.9 (2.5-18.7) ^a^	4.3 (1.3-14.7) ^c^	4.3 (1.2-16.4) ^c^	5.4 (1.3-21.5) ^c^
Gender		5.3 (1.7-17.1) ^b^	5.0 (1.5-16.5) ^b^	4.2 (1.2-14.4) ^c^
Age		1.15 (1.08-1.21) ^a^	1.15 (1.08-1.22) ^a^	1.15 (1.08-1.21) ^a^
BMI		1.05 (0.97-1.14)	1.05 (0.97-1.14)	1.08 (0.99-1.18)
HOMA-IR			1.0 (0.8-1.3)	0.9 (0.7-1.3)
hsCRP				0.9 (0.7-1.0)
^a ^p ≤ 0.001	^b ^p ≤ 0.01	^c ^p < 0.05		

Data are given as odds ratio (95% CI) using multiple logistic regression analysis.

As an addition to model 4 we adjusted for smoking, alcohol consumption, OSA relevant medication (benzodiazepines, tricyclic antidepressants and antipsychotics) and hypertension (systolic and diastolic), with none of these significantly altering the OR of having OSA (data not shown). After replacing AHI cut off 5 (events/hour) with AHI cut off 15 as the dependent variable in the model, glucose tolerance was not significantly associated with AHI ≥ 15.

We tested for correlations between HOMA-IR, hsCRP and glucose tolerance using Spearmans correlation coefficient. HOMA-IR and glucose tolerance had a correlation coefficient of 0.33, p < 0.001. This means a moderate correlation between HOMA and glucose tolerance status, with variation in HOMA-IR explaining 11% of the variation in glucose tolerance. Thus we can safely adjust for HOMA-IR in the logistic regression model. HsCRP and glucose tolerance had a correlation coefficient of 0.03, p = 0.698

PreDM and T2DM were assembled in one group; abnormal glucose tolerance; and replaced with the glucose tolerance categories in the multiple logistic regression model. In model 4 subjects with abnormal glucose tolerance had an OR (95% CI) of having OSA of 4.4 (1.4, 13.8).

Glucose tolerance as a categorical variable was replaced with HbA1c in supplementary logistic regression analyses. HbA1c was not significantly associated with OSA in these models, odds ratio (95% CI) 1.29 (0.79, 2.12, p = 0.314).

## Discussion

This study demonstrates that extremely obese patients with type 2 diabetes and prediabetes have higher odds of OSA, even after adjustment for age, gender, overall obesity (BMI), insulin resistance, inflammation, hypertension, smoking, alcohol consumption and medication.

### Interpretation

That OSA may cause or worsen glucose tolerance has been firmly established over the last few years [7,17-20,34,35]. Our main objective was, however, to explore the reverse relationship by examining whether extremely obese subjects with abnormal glucose tolerance (preDM or T2DM) had higher odds of OSA. In accordance with Foster et al, we have demonstrated a particularly high prevalence of OSA among severely obese patients with T2DM (78% in our study as compared to 86% in the Foster study) [34]. We extend these findings by showing that whilst 2 out of 3 obese patients with preDM had OSA, only 1 out of 3 patients with NGT had OSA. Interestingly, the relationship between glucose tolerance category and OSA was particularly pronounced in premenopausal subjects (figure 4). While less than 20% of the premenopausal patients with NGT had OSA, more than half the patients with abnormal glucose tolerance had OSA. This finding might be a result of the redistribution of adipose tissue to more central parts of the body caused by the hormonal changes during menopause [36]. In older males the prevalence and severity of OSA might decrease because of redistribution of body fat from the central to the peripheral depots. This is possibly a result of lower testosterone levels. Taking into account the very high prevalence of OSA across glucose categories among morbidly obese men and postmenopausal women, the power of the present study is insufficient to reveal any possible differences between glucose categories. In our study less than one third of patients (29%) had NGT, while 42% suffered from preDM. Two out of three patients with preDM had concomitant OSA. In the light of these figures a considerable proportion of extremely obese subjects could suffer from unrecognized OSA.

The relationship between abnormal glucose tolerance (preDM/T2DM) and OSA might have several explanations. First, it is well established that OSA is associated with insulin resistance and high insulin levels [37]. A causal relationship between these conditions remains, however, to be established [38].

As subjects with abnormal glucose tolerance often have high insulin levels, our results are indirectly supported by a prospective study which demonstrated that high insulin levels were associated with higher incidence of sleep apnea [17]. In women with polycystic ovary syndrome (PCOS) insulin resistance is the strongest predictor of OSA [39]. As insulin resistance seems to be the primary defect in these patients, this could support our findings of an association between preDM, T2DM and OSA. One possible mechanism for these observations may be that the inflammation associated with hyperinsulinemia, insulin resistance and visceral adiposity induces OSA [40]. Inflammation of the upper airways (UA) contributes to narrowing the lumen and thus increases the obstructive tendency.

Although the patients with preDM and T2DM had higher HOMA-IR than those with NGT, adjustments for HOMA-IR did not substantially alter the relationship between glucose tolerance category and OSA. This suggests that insulin resistance cannot explain the relationship between glucose intolerance and OSA in our study.

On average the patients with OSA had significantly lower levels of leptin than those without OSA (table 1). As men generally tend to have lower leptin levels than women, the high proportion of men with OSA might partly explain this difference [41]. There was, however, no gender significant difference between the patients with or without OSA in terms of their leptin levels (data not shown). It cannot be ruled out that this is due to a type-II error, encountered because of the low frequency of males without OSA (n = 7).

Our finding of an apparently inverse relationship between leptin and OSA is still to be explored. Some previous studies found a positive correlation between leptin levels and OSA [42,43], whilst others have suggested that the apparent association between OSA and leptin levels is explained by higher BMI [6,11]. By contrast, in our study body weight did not differ significantly between patients with or without OSA.

The pathophysiology of OSA is multi-factorial, the most obvious factor being obesity which is associated with the physical narrowing of the airways. Both adipose tissue and diabetes are known to produce inflammation. Increased mucosal thickness, secondary to general inflammation, may contribute to airway narrowing and collapse. Moreover, diabetic autonomic neuropathy can be a functional factor in OSA by reducing the effectiveness of the UA dilator muscles [15]. This theory is strengthened by the fact that respiratory disturbances are more frequent during rapid eye movement (REM) sleep when the tone of the UA dilator muscles is reduced. The autonomic contribution to maintaining a patent airway is therefore substantial. Accordingly, diabetes could increase the risk of developing OSA via two mechanisms; by inflammation and by impairment of the autonomic nervous system controlling the UA dilator muscles. As well as being a risk factor for OSA, visceral obesity plays an important role in the development of T2DM by mobilizing free fatty acids and inflammatory cytokines, both of which promote insulin resistance [44].

### Strengths and limitations

The strengths of the present study include the relatively high number of extremely obese subjects with a high prevalence of abnormal glucose tolerance and OSA, thereby combining three profoundly intercorrelated medical conditions. The method used to categorise sleep apnea has been validated in several studies. There are some limitations to this study, the main being the cross-sectional study design which eliminates the ability to determine causality. The study includes mainly Caucasians, and as such the findings might not be generalizable to other populations. Our subjects are extremely obese and our results are therefore not directly applicable to less obese or normal weight subjects.

Finally, portable unattended sleep polygraphy was used to give sleep registrations: when compared to full polysomnography this monitor does not give information about sleep stages and thus cannot differentiate between REM and NREM OSA. Exact sleep time was thus not registered through an EEG, and time in bed was used to estimate sleep time.

### Conclusion/clinical implications

In the present study we have shown that preDM and T2DM are commonly observed and associated with OSA in extremely obese subjects. Our findings support the recommendations from the International Diabetes Federation (IDF) suggesting that subjects with T2DM should be screened for OSA [7]. If the relationship between preDM and OSA is verified by others, this may indicate that obese subjects with preDM should also be screened for OSA.

## Abbreviations

AHI: apnea-hypopnea index; BMI: body mass index; CRP: c-reactive protein; IDF: International Diabetes Federation; MOBIL study: morbid obesity treatment, bariatric surgery versus intensive lifestyle intervention study; NGT: normal glucose tolerance; OPG: osteoprotegerin; OSA: obstructive sleep apnea; PreDM: pre-diabetes; REM: rapid eye movement; T2DM: type 2 diabetes mellitus; UA: upper airway; WC: waist circumference; WHR: waist-hip ratio;

## Competing interests

The authors declare that they have no competing interests.

## Authors' contributions

JMF contributed with acquisition of data, statistical analysis and interpretation of data, drafted the manuscript and revised it critically in terms of academic content. JROI contributed to the conception and design of the study and also revised the manuscript critically in terms of academic content. TO contributed to interpretation of data, was involved in drafting the manuscript and revised it critically in terms of academic content. DH contributed to interpretation of data, was involved in drafting the manuscript and revised it critically in terms of academic content. JROI contributed to the statistical analyses, interpretation of data, was involved in drafting the manuscript and revised it critically in terms of academic content. KMV contributed to acquisition of data and revised the manuscript critically in terms of academic content. JH contributed to the conception and design, interpretation of data, was involved in drafting the manuscript and revised it critically in terms of academic content. All authors read and approved the final manuscript.
